# Immune checkpoint inhibitor-related adrenal hypofunction and Psoriasisby induced by tislelizumab: A case report and review of literature

**DOI:** 10.1097/MD.0000000000037562

**Published:** 2024-03-22

**Authors:** Yisi Deng, Manling Huang, Runpei Deng, Jun Wang

**Affiliations:** a The First Clinical Medical School of Guangzhou University of Chinese Medicine, Guangzhou, Guangdong, P.R. China; b Department of Urology, The First Affiliated Hospital of Guangzhou University of Chinese Medicine, Guangzhou, Guangdong, P.R. China; c Nanjing University of Chinese Medicine, Nanjing, Jiangsu, P.R. China.

**Keywords:** adrenal insufficiency, bladder cancer, immune-related adverse events, psoriasis, tislelizumab

## Abstract

**Rationale:**

Immune-related adverse events following treatment with immune checkpoint inhibitors can affect almost every organ. Tislelizumab, a novel humanized Ig G4 programmed death receptor 1 inhibitor, was started for bladder cancer in 2019, but the adverse effects of this drug may not yet be known due to its short time on the market, and there are still some clinical safety concerns. There are few reports of adrenal insufficiency after tislelizumab treatment, which is easily missed, misdiagnosed and life-threatening.

**Patient concerns:**

A 67-year-old male with bladder cancer who developed rash, water-sodium retention, electrolyte disturbances, hypoalbuminemia, low-grade fever, nausea and vomiting, and fatigue after 2 cycles of tislelizumab.

**Diagnosis:**

Immune checkpoint inhibitor-related adrenal hypofunction and Psoriasisby.

**Interventions:**

Suspended tislelizumab treatment and continued glucocorticoid therapy.

**Outcomes:**

The patient showed significant improvement in the above symptoms. But bladder cancer reemerged at the same site.

**Conclusions:**

The advent of immune-related adverse events has increased the complexity of the application of tislelizumab in the treatment of bladder cancer and further research is needed to develop the best treatment guidelines. Early diagnosis and treatment are crucial since the adverse events could endanger lives.

## 1. Introduction

Bladder cancer (BCa) remains the most common malignancy of the urinary tract, with 573,278 patients diagnosed and 212,536 deaths worldwide in 2020.^[1]^ Immune checkpoint inhibitors (ICIs) alone or in combination with other treatments is a treatment option for advanced BCa.^[2]^ Tislelizumab, a humanized recombinant anti-PD-1 monoclonal antibody, has been proven to have good antitumour effects and better tolerability in patients with advanced solid tumors and was approved for marketing in China in December 2019.^[3,4]^ Few adverse reactions of tislelizumab have been reported.^[5]^ The common adverse reactions include fatigue, rash, hypothyroidism and elevated aminotransferase. The incidence of adrenal insufficiency (AI) is approximately 0.2%.^[6]^ There have been no reported cases of AI combined with psoriasis in BCa patients treated with tislelizumab, and the clinical presentation of AI is nonspecific, making it easy to be missed, misdiagnosed, thus endangering patients’ lives. In this case, the lump of patient’s bladder was under control after using tislelizumab, but he firstly developed more and more serious rash. And after 2 cycles of tislelizumab, he developed low-grade fever, nausea and vomiting, fatigue, and electrolyte disturbances, etc. Finally, he was diagnosed with AI by examination of adrenal function and hypophysis. By glucocorticoid therapy, the patient showed significant improvement in the above symptoms. This rare case provides management measures for the use of tislelizumab in BCa complicated by AI and psoriasis, including a thorough examination of adrenal function, early differential diagnosis and prompt glucocorticoid therapy, with the goal of drawing clinicians’ attention to associated adverse effects.

## 2. Case presentation

**Figure 1.**
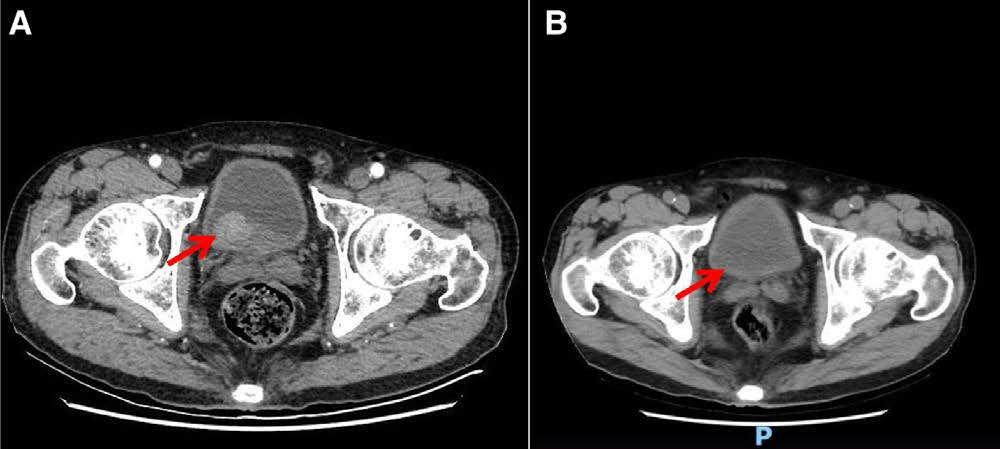
Images of the patient throughout the treatment. (A) Before treatment, abdomen CTU showed localized invasion of the right wall lump of the bladder into the surrounding adipose tissue. (B) After maximal TURBT combined with tislelizumab, abnormal signals was seen on abdomen enhanced CT. CTU = computed tomography urography, TURBT = transurethral resection of the bladder tumor.

**Figure 2.**
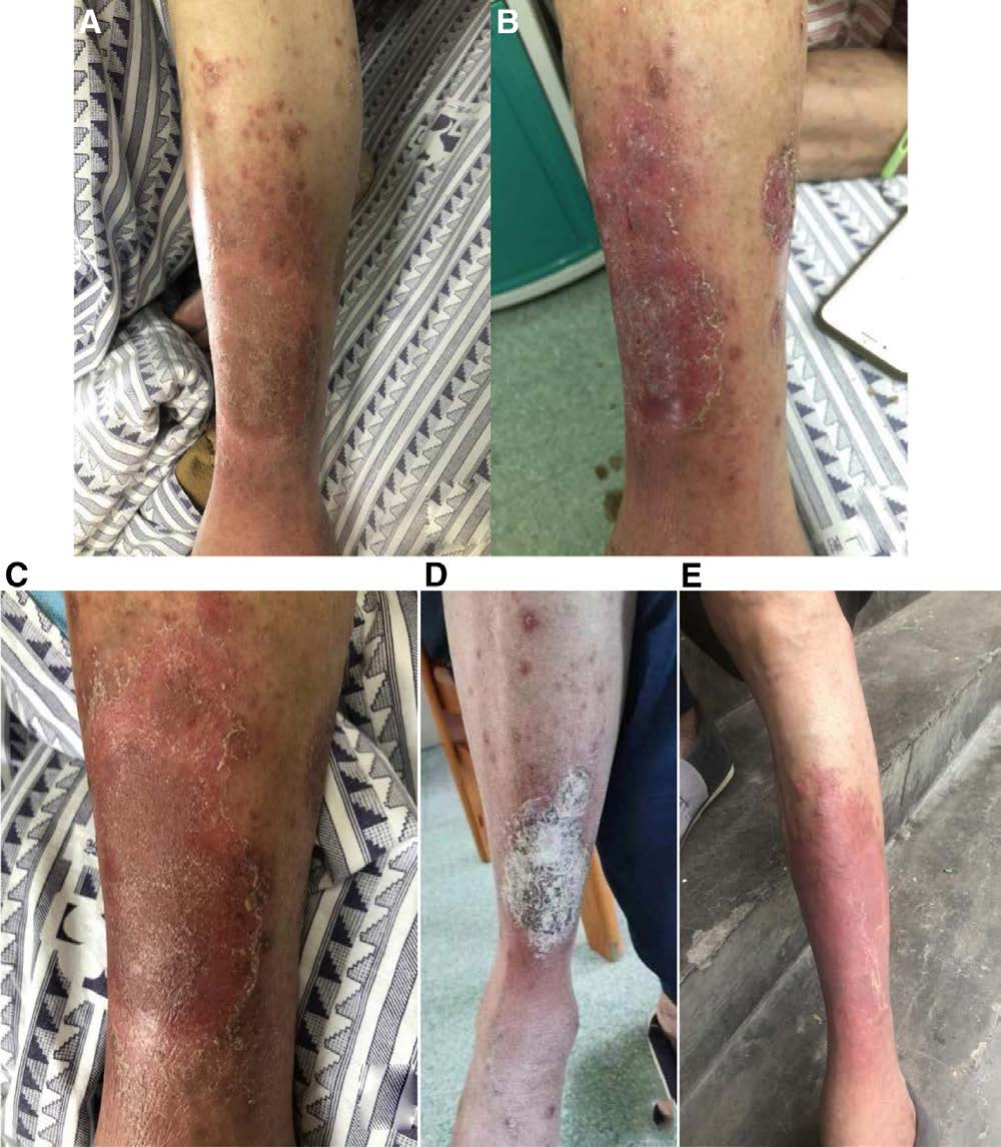
Images of the patient throughout the treatment of psoriasis. (A) The patient returned to the hospital with a rash on February 2022. (B) The patient’s rash improved on May 6, 2022. (C) The patient came to the dermatology department on May 25, 2022 for treatment of psoriasis. (D and E)The patient was discharged after being treated for an improving rash on June 2022.

**Figure 3.**
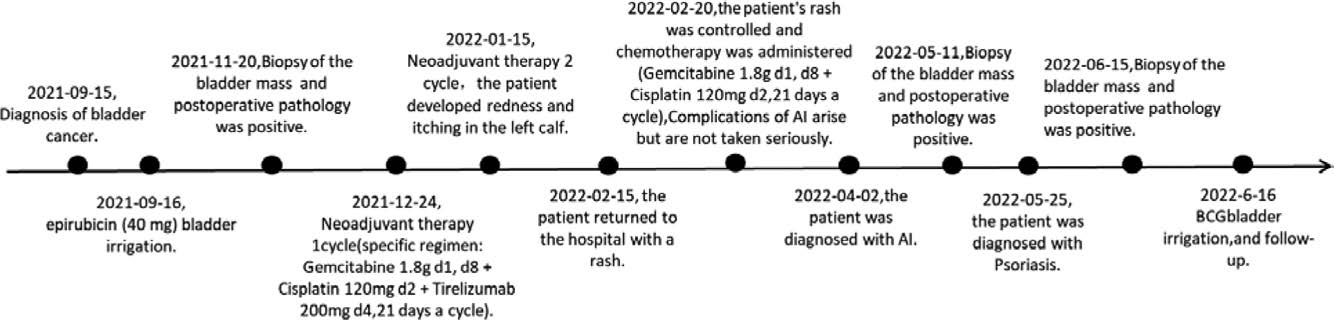
Timeline of disease and treatment.

An Asian male patient, 67 years old, presented to the hospital in September 2021 with “gross hematuria” and a mass in the posterior bladder wall revealed by computed tomography urography (Fig. 1A). Transurethral resection of the bladder tumor was performed on September 15, 2021. Postoperative pathological findings: invasive high-grade uroepithelial cell carcinoma, which was treated with epirubicin (40 mg) bladder irrigation immediately after surgery (once a week for 3 months after surgery). A biopsy of the bladder was performed on December 20, 2021. Postoperative pathological findings: high-grade invasive uroepithelial carcinoma, infiltrating the lamina propria of the mucosa, without invasion of the muscularis. Because of the strong desire to preserve the bladder, the patient was advised to undergo neoadjuvant therapy (specific regimen: gemcitabine 1.8 g d1, d8 + cisplatin 120 mg d2 + tirelizumab 200 mg d4, 21 days a cycle). The first session of treatment went well, with no significant adverse effects. On January 15, 2022, a second phase of neoadjuvant therapy was administered with the same treatment protocol as before, during which the patient developed redness and itching in the left calf, which was subsequently treated with loratadine tablets. On February 15, 2022, the patient returned to the hospital with a rash (Fig. 2A). The dermatology department considered the possibility of psoriasis; while the oncology department considered the possibility of immune-related skin toxicity and recommended suspending tislelizumab. After symptomatic treatment, the patient’s rash was controlled and chemotherapy was administered on February 22, 2022 (gemcitabine 1.8 g d1, d8 + cisplatin 120 mg d2, 21 days a cycle). During treatment, the patient developed water–sodium retention, electrolyte disturbances, hypoalbuminemia, low-grade fever, nausea and vomiting, and fatigue, etc. The patient’s symptoms did not improve well after symptomatic treatment. Adrenal function tests were completed on April 2, 2022 and cortisol was < 0.1 µg/dL, under such circumstances, adrenal insufficiency was considered and treatment with oral methylprednisolone (8 mg Qd) was started on April 3, 2022. Afterwards, the patient showed significant improvement in the above symptoms. Cortisol: 0.143 µg/dL and adrenocorticotropic hormone (ACTH) < 1pg/mL on April 6, 2022; the electrolytes and serum albumin were checked and were normal. On May 6, 2022, the patient was admitted to the hospital for review. The patient’s rash improved (Fig. 2B), cortisol was <0.1 µg/dL and adrenocorticotropic hormone was <1 pg/mL, and treatment with oral methylprednisolone (8 mg Qd) was continued. The bilateral adrenal gland on the CT abdomen showed no abnormalities, and the bladder mucosa and surrounding tissue in the surgical area were more significantly enhanced (Fig. 1B). No abnormality in pituitary gland on magnetic resonance imaging. A biopsy of the bladder mass was performed on May 11, 2022 and the postoperative pathology showed a high-grade invasive uroepithelial carcinoma at the “base of the bladder.” He came to the dermatology department on May 25, 2022 for treatment of psoriasis and was discharged after being treated for an improving rash (Fig. 2C–E). A biopsy of the bladder mass was performed on June 15, 2022, which suggested high-grade invasive uroepithelial carcinoma with no invasion of the muscular layer. The bladder was subsequently perfused with Bacille Calmette-Guérin vaccine(once a week for 8 weeks) and no significant signs of recurrence were seen on cystoscopy, which was continued in outpatient follow-up (Fig. 3). The study was approved by the Ethics Committee of The First Affiliated Hospital of Guangzhou University of Chinese Medicine (NO. JY2023-142). Informed consent was obtained from the patient.

## 3. Discussion

BCa is one of the most common malignancies in urology, ranking 10th in terms of incidence and 13th in terms of mortality among systemic malignancies.^[1]^ In recent years, more and more ICIs have been used in the neoadjuvant treatment of high-grade uroepithelial carcinoma. A meta-analysis found that advanced or metastatic cancer patients have better progression-free survival and overall survival with the help of programmed death receptor 1 (PD-1)/PD-L1 inhibitors than conventional treatment or placebo, and a higher safety profile.^[7]^ As with the introduction of any new therapy, the activation of the immune system mediated by ICIs therapy can involve normal tissues and organs, resulting in immune-related adverse events (irAEs).^[8]^ In Phase III trials of PD-1/PD-L1 inhibitors, the incidence of irAEs was as high as 30%, with severe irAEs ranging from 0.3% to 13%.^[9]^ Common adverse effects concerning treatment with tislelizumab are: elevated transaminases, rash, fatigue; while irAEs are rare and consist primarily of: hypothyroidism/hyperthyroidism, skin adverse reactions, hepatitis, pneumonia, colitis, thyroiditis. If irAEs occur, they may be temporarily or permanently discontinued.^[6]^ Multi-organ irAEs including pneumonia, myocarditis, hepatitis and secondary AI due to hypophysitis have been reported after 1-cycle treatment with tislelizumab in non-small cell lung cancer.^[10]^

A randomized controlled trial (RCT) of tislelizumab as treatment for advanced or metastatic esophageal squamous cell carcinoma suggested that the reported rate of treatment-related adverse events (TRAEs) was 73.3%, The most common TRAEs were increased aspartate aminotransferase (11.4%), anemia (11.0%), and hypothyroidism (10.2%), no AI and immune rash was reported.^[11]^No RCTs of tislelizumab in BCa have yet been published. Comprehensive irAE data on tislelizumab are still being collected and analyzed. Data from a single-arm phase 2 trial of tislelizumab in patients with locally advanced or metastatic urothelial carcinoma suggested that the reported rate of irAEs was 27%. The most common irAEs were skin adverse reaction (12%), hypothyroidism (11%), and hyperthyroidism (6%) and AI was no reported.^[12]^ Whereas, tislelizumab in the treatment of advanced or metastatic uroepithelial carcinoma, anemia and fever are the most common adverse reactions, and the most common irAEs are cutaneous adverse reactions (12%), hypothyroidism (11%), hyperthyroidism (6%), with the incidence of rash at approximately 3% and no reported AI.^[13]^ The interesting thing is that the occurrence of irAEs is positively correlated with objective disease remission, progression-free survival, and overall survival, while irAEs of grade 3 and higher have better objective remission rates but worse overall survival.^[14]^ In this case, the patient developed rash, fatigue, weakness, weight loss, electrolyte disturbances and hypoproteinemia after 2 cycles of treatment with tirelizumab, after excluding possible factors such as autoimmune hypophysitis, long-term oral glucocorticoid use, surgery and trauma, and no literature reports of AI due to chemotherapy (cisplatin and gemcitabine) were retrieved. The possibility of AI due to immunotherapy with tirelizumab was considered in conjunction with the patient’s concurrently reduced ACTH and cortisol (Table 1).

**Table 1:** Naranjo scoring criteria and scoring results of adrenal insufficiency caused by tislelizumab.

Domains	Naranjo scoring			Ttislelizumab		
	Yes	Not	Not sure or N/A	Yes	Not	Not sure or N/A
1. Was there an improvement in the main symptom or condition for which the homeopathic medicine was prescribed?	+2	−1	0	+2		
2. Did the clinical improvement occur within a plausible timeframe relative to the drug intake?	+1	−2	0			0
3. Was there an initial aggravation of symptoms?	+1	0	0	+1		
4. Did the effect encompass more than the main symptom or condition (i.e., were other symptoms ultimately improved or changed)?	+1	0	0	+1		
5. Did overall well-being improve? (suggest using validated scale)	+1	0	0		0	
6A Direction of cure: did some symptoms improve in the opposite order of the development of symptoms of the disease?	+1	0	0		0	
6B Direction of cure: did at least 2 of the following aspects apply to the order of improvement of symptoms: –from organs of more importance to those of less importance? –from deeper to more superficial aspects of the individual? –from the top downwards?	+1	0	0		0	
7. Did “old symptoms” (defined as nonseasonal and noncyclical symptoms that were previously thought to have resolved) reappear temporarily during the course of improvement?	+1	0	0		0	
8. Are there alternate causes (other than the medicine) that—with a high probability—could have caused the improvement? (Consider known course of disease, other forms of treatment, and other clinically relevant interventions)	−3	+1	0	+1		
9. Was the health improvement confirmed by any objective evidence? (e.g., laboratory test, clinical observation, etc.)	+2	0	0	+2		
10. Did repeat dosing, if conducted, create similar clinical improvement?	+1	0	0			0

*Note*: Maximum score = 13, minimum score = -6, the judgment result is divided into 4 levels, which is “im-possible” (total score ≤ 0), “possible” (total score 1–4), “very likely” (total score 5–8), “determined” (total score ≥ 9 points).

AI is a rare but serious adverse after treatment with ICIs. It occurs over an indeterminate period of time, ranging from a month to several years, and the lack of specificity of clinical symptoms makes it easy for physicians to overlook. If AI is unrecognized, untreated or misdiagnosed, it will progress to an adrenal crisis, which can be life-threatening. One study found a 0.7% chance of AI after using ICIs, and only a 0.2% chance for grades 3 and higher.^[15]^The pathogenesis of AI associated with ICIs is not properly understood. With respect to elevated levels of 21-hydroxylase and adrenal cortex antibody titers found in a patient treated with pembrolizumab immunotherapy for metastatic choroidal melanoma, it remains unclear whether adrenocortical antibodies play a role in the pathogenesis and prognosis of ICIs-related AI.^[16]^ Symptoms and signs of AI are nonspecific, which may include abdominal discomfort, anorexia, fatigue, drowsiness, muscle cramps and discomfort, nausea, postural dizziness, salt cravings, and vomiting.^[17]^ Adrenal crisis will occur when AI is not promptly recognized, which can be life threatening. Adrenal crisis is characterized by severe weakness, fainting, nausea, vomiting, abdominal pain, confusion, altered mental status, and delirium, which can progress to shock and death if left untreated. In this case, the patient suffered from fatigue, nausea, vomiting, low sodium, low protein and other discomfort occurred, which was easily confused with the bodily functions consumed by tumor diseases. Due to the lack of visible AI and the lack of experience with treatment, treatment was delayed. Fortunately, the patient was finally diagnosed in time. Therefore, patients treat with tislelizumab for the first time and inexperienced young clinicians should always pay attention to the occurrence of AI.

In addition, it is necessary to identify central adrenal insufficiency (CAI) and the primary adrenal insufficiency (PAI). CAI can be caused by ICIs-related hypophysitis or pituitary metastasis but no abnormalities were found in the pituitary gland in this case. PAI may be caused by ICIS-related PAI, bilateral adrenal metastasis or bilateral adrenal hemorrhage, resulting in destruction and/or damage to the adrenal cortex. PAI requires mineralocorticoid and glucocorticoid replacement therapy, but CAI does not because the adrenal zona glomerulosa that synthesize aldosterone in CAI remain intact and the production of mineralocorticoid is preserved. While hypoglycemia and hyperkalemia are relatively uncommon, hyponatremia and hyperkalemia are common in PAI due to concurrent glucocorticoid and mineralocorticoid deficiencies. Patients with PAI tend to develop hyperpigmentation, while those with secondary AI tend to have pale skin.^[18]^ Abdominal imaging of ICIs-associated AI may show evidences of adrenalitis, presenting bilateral enlarged adrenals with relatively smooth borders,^[19]^ but the imaging results have also been reported to be normal.^[16]^

According to the severity of AI, there are grades 1 to 4, with grades 1 to 2 not requiring interruption of ICIs therapy and grades 3 to 4 requiring the interruption process. Glucocorticoid replacement therapy is a mainstay treatment for AI, with different doses depending on the severity of symptoms. For patients with symptoms or signs of adrenal insufficiency, baseline ACTH and cortisol measurements can be obtained prior to corticosteroid administration and, if safe, can be used for diagnostic purposes. However, empirical treatment with corticosteroids should not be delayed in acutely ill patients with clinical symptoms and signs of adrenal insufficiency. In the acute phase, hydrocortisone 100 mg can be administered intravenously, followed by the further 50 mg every 6 to 8 hours, after which the treatment is switched to oral hydrocortisone (10–20 mg in the morning and 5–10 mg in the afternoon),^[20]^ or hydrocortisone 10 to 30 mg daily (divided into 2 oral doses), fludrocortisone 0.05 to 0.2 mg daily.^[21]^ Two cases of tislelizumab combined with chemotherapy for gastric cancer were reported, with AI after 6 and 13 cycles of treatment respectively, which improved with hydrocortisone (10 mg twice daily).^[22]^ In accordance with the Endocrine Society guidelines on AI, we recommend that physicians counsel all patients with AI on sick leave rules, stress doses, and emergency corticosteroid administration, instruct them to receive a medical alert device (such as a handedness or necklace) about AI, and prescribe an injectable package for the emergency use of high-dose corticosteroids.^[17]^ Long-term outcomes of AI related to ICIs have not been reported in any studies, however, according to 2 patients with ICIs-related AI, it may be irreversible in patients with adrenal insufficiency caused by ICIs therapy, requiring long-term glucocorticoid and mineralocorticoid replacement therapy.

Adverse skin reactions are the earliest complication following treatment with ICIs. The exact mechanism for the development of psoriasis-like drug rash following treatment with ICIs is unknown and may be that ICIs enhance the activity of helpers T-cell 1 and T-cell 17, resulting in the production of interleukin-17.^[23]^ Herpetic aspergillosis, vitiligo-like skin hypopigmentation/depigmentation and psoriasis-like rashes are often seen after immunotherapy with PD-1/PD-L1 inhibitors.^[23]^ A Korean retrospective analysis found that melanoma and genitourinary malignancies were most likely to experience cutaneous adverse reactions following the use of PD-1/PD-L1 inhibitors; pruritic, psoriatic, and urticaria are the most common types of rash, with median times of appearance of 15, 20, and 6.5 weeks respectively; urticaria appeared earlier after immunotherapy, while keratoacanthoma appeared later.^[24]^

A retrospective analysis of tislelizumab in combination with gemcitabine plus cisplatin as first-line treatment for locally advanced or metastatic BCa suggested that the most common TRAEs were anemia (57.1%), decreased appetite (57.1%), nausea/vomiting (50.0%), and thyroid disorders (42.9%). The reported rate of rash was 14.3%.^[12]^ Another study found that patients with a previous history of psoriasis developed psoriasis-like rashes earlier with PD-1 inhibitors.^[25]^ Main treatment for immune-associated rashes is: topical corticosteroids for mild to moderate rashes (grades 1–2), systemic corticosteroids for severe rashes (grade 3) and discontinuation of immunotherapy for grade 4 rashes.^[23]^ Most psoriasis-like rashes can be controlled by topical steroids and/or vitamin D analogues. In contrast, drug-resistant psoriasis can be treated with retinoids (retinoic acid) or immunomodulatory biologics (methotrexate), and light therapy (narrow-band ultraviolet B light therapy) is also useful (except for patients with melanoma).^[23,25]^ There are few reports of adverse skin reactions following the use of tislelizumab. There is only one report of pemphigus herpetiformis after 6 courses of tislelizumab for non-small cell lung cancer, and the rash improved after the administration of intravenous methylprednisolone (40 mg/day), followed by oral prednisone (40 mg/day) for maintenance treatment.^[26]^ It has also been shown that the presence of cutaneous adverse reactions (other than mucositis and hyperhidrosis) following the use of PD-1/PD-L1 inhibitors is an indicator of effective treatment and a good prognosis, and that the earlier the adverse reaction occurs, the lower the risk ratio is.^[27]^ In retrospect, the patient started to develop a psoriasis-like rash on the left lower leg after 2 cycles of tislelizumab, which gradually progressed to the whole body and finally developed typical psoriatic lesions; the rash improved after treatment with main glucocorticoids (hydrocortisone butyrate cream, dexamethasone ointment, halometasone cream, tretinoin acetate solution, prednisone acetate tablets), vitamin D3 preparations (tacalcitol ointment) and infection prophylaxis (mupirocin ointment). However, this case is not based on systematic studies, the information and evidence provided are limited, so more RCTs are needed to study.

## 4. Conclusions

BCa is a malignancy with a high recurrence rate, and immediate postoperative, induction and maintenance bladder perfusion therapy and postoperative follow-up are essential, especially for patients with non-muscle invasive BCa. Tislelizumab, a novel immunotherapy, has been used in the neoadjuvant treatment for non-muscle invasive BCa, and several clinical trials have demonstrated its significant anti-tumor effects. As ICIs become more widely used in clinical practice, their adverse effects will gradually become apparent. There are few reports of AI after tislelizumab treatment for BCa, which is easily missed and misdiagnosed in clinical practice, and there is a risk of life-threatening adrenal crisis. Therefore, clinical attention should be paid to the occurrence of AI to ensure the safety of drug administration when we choose tislelizumab treatment.

## Acknowledgments

The authors would like to thank all people involved in this work.

## Author contributions

**Methodology:** Jun Wang.

**Writing – original draft:** Yisi Deng.

**Writing – review & editing:** Yisi Deng, Manling Huang, Runpei Deng.
